# Neoadjuvant chemotherapy followed by concurrent chemoradiotherapy versus concurrent chemoradiotherapy followed by adjuvant chemotherapy in locally advanced nasopharyngeal carcinoma

**DOI:** 10.1186/s12885-018-4210-3

**Published:** 2018-03-27

**Authors:** Jiraporn Setakornnukul, Kullathorn Thephamongkhol

**Affiliations:** Division of Radiation Oncology, Department of Radiology, Faculty of Medicine, Siriraj Hospital, Mahidol University, 2 Wanglang Road, Bangkoknoi, Bangkok, 10700 Thailand

**Keywords:** Neoadjuvant chemotherapy, Adjuvant chemotherapy, Concurrent chemoradiotherapy, Locally advanced nasopharyngeal carcinoma, Intensity-modulated radiotherapy

## Abstract

**Background:**

Concurrent chemoradiotherapy followed by adjuvant chemotherapy (CCRT-AC) is currently recommended as the standard treatment for locally advanced nasopharyngeal carcinoma (LA-NPC). Neoadjuvant chemotherapy followed by concurrent chemoradiotherapy (NAC-CCRT) is an alternative strategy for decreasing tumor size and controlling micrometastases before main treatment. The aim of this study was to investigate and compare survival outcomes between LA-NPC patients treated with CCRT-AC and those treated with NAC-CCRT.

**Methods:**

This retrospective cohort study included consecutive histologically confirmed LA-NPC patients that were treated with NAC-CCRT or CCRT-AC at Siriraj Hospital during the March 2010 to October 2014 study period. CCRT in both protocols consisted of 3-week cycles of cisplatin 100 mg/m^2^ with concurrent radiotherapy. Either NAC or AC consisted of 3-week cycles of cisplatin on day 1 and fluorouracil/leucovorin on days 1–4 for a maximum three cycles. The primary endpoint was 5-year overall survival (OS). Flexible parametric survival analysis was used, because the proportional hazards assumption of Cox regression was violated.

**Results:**

Of the 266 LA-NPC patients that received treatment during the study period, 79 received NAC-CCRT and 187 received CCRT-AC. Median follow-up was 37 months. Significantly more patients with advanced clinical stage (stage IVA-IVB) received NAC-CCRT (86% in NAC-CCRT vs. 29% in CCRT-AC; *p* < 0.001). Compared to CCRT-AC in crude analysis, 3-year and 5-year OS of NAC-CCRT were 72% vs. 86% and 62% vs. 75% respectively (*p* = 0.059). Interestingly, the 3-year and 5-year post-estimation adjusted OS was 84% and 74% for NAC-CCRT and 81% and 70% for CCRT-AC, respectively (HR: 0.83, 95% confidence interval (CI): 0.45–1.56; *p* = 0.571). Also, adjusted analysis of distant-metastasis survival, NAC-CCRT showed HR was 0.79 (95% CI:0.37–1.72, *p* = 0.557). Conversely, adjusted analysis of locoregional relapse (LLR)-free survival revealed NAC-CCRT to have a significantly higher risk of LRR (HR: 2.18, 95% CI: 0.98–4.87; *p* = 0.057).

**Conclusions:**

The results suggested that prognosis in the NAC-CCRT treated patients was not superior to that of the CCRT-AC treated individuals. In patients that receive neoadjuvant chemotherapy, locoregional relapse should be of concern. High-risk distant metastasis patients (N3 stage) that could achieve survival advantage from NAC-CCRT is an interesting and important topic for further study.

**Electronic supplementary material:**

The online version of this article (10.1186/s12885-018-4210-3) contains supplementary material, which is available to authorized users.

## Background

Nasopharyngeal carcinoma is the most common head and neck cancer in Asia. The virus associated with nasopharyngeal carcinoma is Epstein-Barr virus (EBV), which is frequently associated with differentiated non-keratinizing carcinoma (type 2) and undifferentiated non-keratinizing carcinoma (type 3) according to World Health Organization (WHO) classification [1–3]. These types of pathology are usually found in endemic areas, and their prognosis is poorer than keratinizing type nasopharyngeal carcinoma [4]. Peng, et al. reported that EBV-positive patients had a higher rate of distant metastasis than EBV-negative patients (15% vs. 3.6%, respectively). As a result, 4-year distant metastasis-free survival (DMFS), disease-free survival (DFS), and overall survival (OS) were lower in EBV-positive patients than in EBV-negative patients (85% vs. 97%; 77% vs. 89%; and, 86% vs. 94%, respectively) [5]. Taken together, patients with non-keratinizing nasopharyngeal carcinoma are at higher risk for developing distant metastasis.

Radiotherapy is mandatory treatment for nasopharyngeal carcinoma due to its inoperable anatomical location and its radiosensitivity. For locally advanced nasopharyngeal carcinoma (LA-NPC), combined chemotherapy and radiotherapy improved overall survival compared to radiotherapy alone, with a 6.3% absolute benefit observed at 5 years according to MAC-NPC meta-analysis [6]. Intergroup study 0099 reported that concurrent chemoradiotherapy followed by adjuvant chemotherapy (CCRT-AC) delivered a significantly better 5-year overall survival benefit than radiotherapy alone (67% vs. 37%, respectively) [7]. The efficacy of CCRT-AC regimen was also studied by Lee, et al. [8, 9] and Wee, et al. [10] in endemic areas.

Distant metastasis is still a major pattern of failure in nasopharyngeal carcinoma, occurring in 13–21% of LA-NPC patients after CCRT-AC in endemic areas (6, 7, 9). Once a patient develops distant metastasis, the objective of treatment will be changed to palliation, and median survival will drop to within one to one and a half years [11]. A high rate of distant metastasis was found predominantly in patients with bulky cervical neck node and in patients with tumor invading the cavernous sinus [12, 13]. In these cases, it has been hypothesized that neoadjuvant chemotherapy followed by concurrent chemoradiotherapy (NAC-CCRT) may be more efficacious than CCRT-AC for controlling micrometastasis, reducing distant metastasis rate, and improving overall survival in some high-risk patients with large cervical lymph node (N3) and in patients with tumor invasion of the cavernous sinus (T4). In support of this hypothesis, Lee, et al. reported a non-significant progression-free survival benefit from NAC-CCRT compared to CCRT-AC with conventional radiotherapy and platinum-based chemotherapy/5-fluorouracil (5-FU) [14]; however, this study was insufficiently powered to detect significant benefit or association due to the small sample size in each arm.

Although both NAC-CCRT and CCRT-AC have been introduced into routine practice, the benefit of neoadjuvant chemotherapy (NAC) or adjuvant chemotherapy (AC) with concurrent chemoradiotherapy (CCRT) has not been conclusively established. Accordingly, the aim of this study was to investigate and compare survival outcomes between LA-NPC patients treated with CCRT-AC and those treated with NAC-CCRT.

## Methods

### Study design

Consecutive patients diagnosed with stage II-IVB nasopharyngeal carcinoma [classification by the 7th edition of the American Joint Committee on Cancer staging system (AJCC)] and treated by radiotherapy with curative intent at Siriraj Hospital during the March 2010 to October 2014 study period were retrospectively reviewed. The inclusion criteria were pathologically confirmed keratinizing, differentiated non-keratinizing, or undifferentiated nasopharyngeal carcinoma (WHO classification), ≥18 years of age, and performance status sufficient to withstand treatment with combined intensity-modulated radiotherapy (IMRT) and platinum-based chemotherapy. Patients with previous cancer diagnosis or who received cancer treatment beyond chemotherapy and radiotherapy were excluded. All included patients received a pretreatment evaluation that included general physical examination; transnasal endoscopy; and, computed tomography (CT) of head and neck or magnetic resonance imaging (MRI) of nasopharynx and neck for primary tumor and nodal staging. Systemic investigation included chest X-ray or CT of chest, ultrasonography or CT of upper abdomen, and bone scan.

### Treatment and follow-up

Simultaneous integrated boost (SIB) technique is commonly used for IMRT at our center, as follows: 70 Gray (Gy) for gross disease; 60–63 Gy for high risk region; and, 54–57 Gy for intermediate risk region, according to RTOG 0615 protocol [15]. The decision regarding treatment regimen between CCRT-AC and NAC-CCRT was decided at tumor board, which consisted of an otolaryngologist, a medical oncologist, and a radiation oncologist. According to protocol at our center, patients with bulky disease such as T4 lesion or N3 disease were offered NAC with platinum-based chemotherapy for three cycles, and then CCRT. Other types of LA-NPC patients were offered CCRT-AC. CCRT in both protocols consisted of 3-week cycles of cisplatin 100 mg/m^2^ with concurrent radiotherapy. Either NAC or AC consisted of 3-week cycles of cisplatin on day 1 and fluorouracil/leucovorin on days 1–4 for a maximum three cycles. Some patients who had renal insufficiency or intolerance to cisplatin were prescribed carboplatin.

After completion of radiotherapy, patients were evaluated for response to treatment with transnasal endoscopy and nasopharyngeal biopsy by otolaryngologist. NPC patients with T3–4 tumor also had follow-up imaging of nasopharynx and neck with CT scan or MRI. For surveillance, routine follow-up in pathologically confirmed complete response was ENT examination every 2–4 month during the first 2 years, then every 4–6 months thereafter. In cases where residual tumor could not be differentiated from post-treatment effect, transnasal endoscopy and CT/MRI scan were repeated for re-evaluation. Patients with no further progressive lesion were defined as complete response.

### Patient characteristics and endpoints

Patient demographic and clinical data that was collected included gender; age; patient factors and histology by WHO classification; TNM staging according to 7th edition of American Joint Committee on Cancer/Union for International Cancer Control (AJCC/UICC) for tumor factors; and, chemotherapy regimen, number of cycles, radiotherapy dose, and overall radiotherapy treatment time (OTT). Treatment compliance data was also collected and analyzed.

The primary endpoint was 5-year overall survival (OS). The secondary endpoints were distant metastasis-free survival (DMFS) and locoregional relapse-free survival (LRRFS). All intervals were calculated from the first date of initial treatment, which was the date of first chemotherapy cycle for NAC-CCRT and the date of first radiotherapy for CCRT-AC. For OS, the last date was date of death from any cause or date of last follow-up. For DMFS, a distant metastasis event was defined as disease recurrence outside the nasopharyngeal region and cervical lymph node. The last date of DMFS was defined as the date of distant metastasis, date of last follow-up, or date of death. For LRRFS, any new pathologically confirmed nasopharyngeal carcinoma tumor occurring in the nasopharynx and/or cervical neck node was defined as locoregional recurrence in complete response patient from prior definitive treatment. In patients that did not achieve complete response, the last date was the date of evidence of primary nasopharyngeal tumor and/or cervical lymph node progression, date of death, or date of loss to follow-up.

### Statistical analysis

To compare baseline characteristics between NAC-CCRT and CCRT-AC, independent t-test was used for continuous variables, and Fisher’s exact test was used for categorical variables. Kaplan-Meier method was used for survival analysis, and our statistical hypothesis was tested by log-rank test for crude analysis and reported as survival curves. Flexible parametric survival analysis was used to perform adjusted survival outcome analysis, due to violation of proportional hazard assumption by Cox regression. Due to the fact that our data did not show time dependent effect, we reported hazard ratio (HR) and 95% confidence interval (95% CI) with *p*-value. The pre-specified adjusted factors were gender, age, histology type, AJCC staging, and completion of radiation treatment. We planned to include all of these factors in multivariate analysis regardless of their level of statistical significance in univariate analysis. Post-estimation from flexible parametric survival was used to construct survival curves. Competing risk Cox regression analysis was done for locoregional relapse-free survival, and subdistribution hazard ratio was reported. All statistical tests were two-tailed, and a *p*-value of less than 0.05 was regarded as being statistically significant.

## Results

During the 2010 to 2014 study period, seventy-nine LA-NPC patients (30%) received NAC-CCRT and 187 LA-NPC patients (70%) received CCRT-AC. The median follow-up time was 37 months. Eighty-six of 266 patients (32%) were lost to follow-up, including 52 patients (28%) in the CCRT-AC group and 34 patients (43%) in the NAC-CCRT group. However, vital status (dead/alive) of these patients could be tracked through the Thailand Office of Central Civil Registration.

The male to female gender proportion was 2:1. Two-thirds of histologic classification was non-keratinizing nasopharyngeal carcinoma, and one-third was undifferentiated nasopharyngeal carcinoma. The NAC group had a far higher proportion of IVA-IVB stage tumor than the AC group (86% vs. 29%, respectively), which was consistent with the treatment policy at our center. The NAC group had more patients with younger age. All other patient characteristics were similar between groups, including gender and histology (Table 1).

**Table 1:** Patient characteristics

Characteristics	NAC-CCRT^a^	CCRT-AC^b^	*p*-value
	*N* = 79	*N* = 187	
Age: Mean (SD)	47.9 (12.6)	51.9 (11.2)	0.011
Min-Max			
Gender: Men	56 (71%)	129 (69%)	0.880
Women	23 (29%)	58 (31%)	
WHO classification: Type I	3 (4%)	4 (2%)	0.640
Type II	51 (65%)	126 (67%)	
Type III	25 (32%)	57 (31%)	
T stage: T1	11 (14%)	53 (28%)	< 0.001
T2	15 (19%)	51 (27%)	
T3	10 (13%)	41 (22%)	
T4	43 (54%)	42 (23%)	
N stage: N0	11 (14%)	24 (13%)	< 0.001
N1	9 (11%)	38 (20%)	
N2	30 (38%)	109 (58%)	
N3	29 (37%)	16 (9%)	
AJCC staging: II-III	11 (14%)	132 (71%)	< 0.001
IVA-IVB	68 (86%)	55 (29%)	

^a^NAC-CCRT: neoadjuvant chemotherapy followed by concurrent chemo-radiotherapy

^b^CCRT-AC: concurrent chemo-radiotherapy followed by adjuvant chemotherapy

All patients who received NAC-CCRT tolerated NAC well, and 73% of them completed 3 cycles of chemotherapy. Only 4 % of patients did not receive concurrent chemotherapy during radiotherapy due to poor performance status after neoadjuvant chemotherapy (Table 2). Regarding concurrent chemotherapy sessions completed, there was no difference in the number of patients who completed three cycles of chemotherapy between patients who received neoadjuvant chemotherapy before and patients who started with concurrent chemoradiotherapy (22% vs. 24%). In contrast, thirty-three patients (17%) who started with concurrent chemoradiotherapy did not receive any adjuvant chemotherapy due to severe mucositis, significant weight loss, and/or performance level that was not high enough for the patient to receive further chemotherapy treatment (Table 2). Per-protocol was analyzed because three patients in NAC-CCRT did not received concurrent chemotherapy and 33 patients in CCRT-AC did not received adjuvant chemotherapy After this exclusion, we found that NAC-CCRT is no longer better than CCRT-AC in terms of overall survival and distant metastases due to less events in CCRT-AC after exclusion patients with poor performance status and intolerance to further adjuvant chemotherapy (Additional file 1: Per-protocol analysis.docx).

**Table 2:** Compliance of radiotherapy and chemotherapy

Treatment	NAC-CCRT^a^	CCRT-AC^b^
	*N* = 79	*N* = 187
Radiotherapy		
Dose: Median (range)	69.96 (0,- 76.3)	69.96 (25.4,- 78.0)
Incomplete radiotherapy	2 (2.5%)	7 (3.7%)
Overall treatment time (days): mean (SD)	52.3 (11.3)	51.4 (9.8)
Chemotherapy		
NAC/AC	NAC	AC
No NAC/AC	–	33 (17%)
< 3 cycles	21 (27%)	32 (17%)
3 cycles	58 (73%)	122 (66%)
CCRT		
No CMT	3 (4%)	–
< 3 cycles	59 (75%)	142 (76%)
3 cycles	17 (22%)	45 (24%)

^a^NAC-CCRT: neoadjuvant chemotherapy followed by concurrent chemo-radiotherapy

^b^CCRT-AC: concurrent chemo-radiotherapy followed by adjuvant chemotherapy

**Fig. 1:**
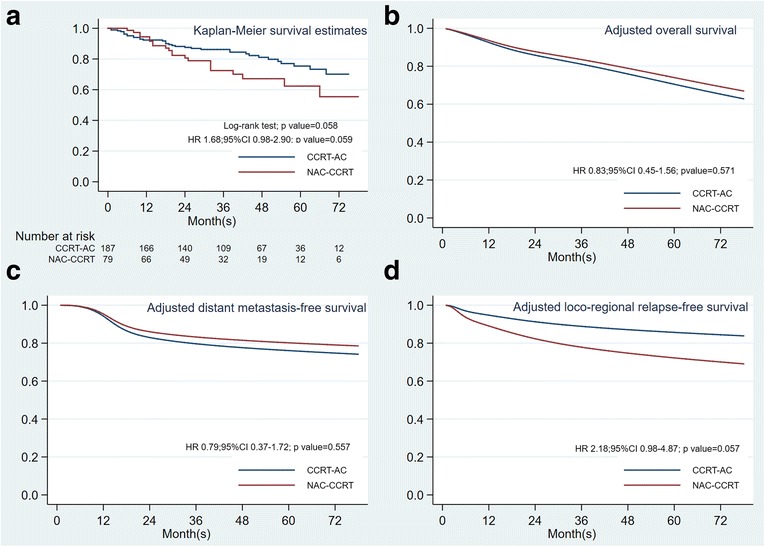
Survival curve comparison between neoadjuvant chemotherapy followed by concurrent chemoradiotherapy (NAC-CCRT) *(Red line)* and concurrent chemoradiotherapy followed by adjuvant chemotherapy (CCRT-AC) *(Blue line)* **a**: Non-adjusted overall survival **b**: Adjusted overall survival **c**: Adjusted distant metastasis-free survival **d**: Adjusted loco-regional relapse-free survival

The 5-year overall survival from all patients in this cohort was 72% (95% confidence interval (CI) 64%–79%). NAC-CCRT showed a non-statistically significance of inferior survival in crude analysis [hazard ratio (HR): 1.68, 95% CI: 0.98–2.90; *p* = 0.059], with 3-year and 5-year overall survival of 72% and 62% for NAC-CCRT, and 86% and 75% for CCRT-AC, respectively (Table 3 and Fig. 1a). However, subgroup analysis by AJCC staging system showed no difference in 3-year overall survival between NAC-CCRT and CCRT-AC, with 100% and 93% OS for stage II-III, and 68% and 71% OS for stage IVA-IVB, respectively. Post-estimation adjusted overall survival analysis showed a non-statistically significance of survival benefit from NAC-CCRT compared to CCRT-AC, with a hazard ratio of 0.83 (95% CI: 0.45–1.56; *p* = 0.571) (Table 3 and Fig. 1b). Interestingly, the post-estimation adjusted 3-year and 5-year overall survival was 84% and 74% for NAC-CCRT and 81% and 70% for CCRT-AC, respectively.

**Table 3:** Main result; overall survival, distant metastasis free survival, loco-regional relapse free survival of the study

Event	NAC-CCRT^a^, *n* (%) vs CCRT-AC^b^, *n* (%)	*p*-value
	Hazard Ratio (95% confidence interval)	
Overall survival		
Dead	21 (27%) vs 35 (19%)	0.187
Univariable analysis	1.68 (0.98–2.90)	0.059
Multivariable analysis^c^	0.83 (0.45–1.56)	0.571
Distant metastasis free survival		
Distant metastasis	13 (16%) vs 30 (16%)	1.000
Univariable analysis	1.26 (0.66–2.42)	0.483
Multivariable analysis^c^	0.79 (0.37–1.72)	0.557
Loco-regional relapse free survival		
Loco-regional failure	19 (24%) vs 18 (10%)	0.003
Univariable analysis	2.88 (1.51–5.50)	0.001
Multivariable analysis^c^	2.18 (0.98–4.87)	0.057

^a^NAC-CCRT: neoadjuvant chemotherapy followed by concurrent chemo-radiotherapy

^b^CCRT-AC: concurrent chemo-radiotherapy followed by adjuvant chemotherapy

^c^Adjusted factors: gender, age, histology type, AJCC staging, complete of radiation treatment

Forty-three patients (16%) developed distant metastasis (DM) at a median time of 34 months. The distant metastatic rate was 16% in both groups. Three-year distant metastatic-free survival (DMFS) was 79% in the NAC-CCRT group, and 84% in the CCRT-AC group (*p* = 0.483). Adjusted analysis by flexible parametric survival model revealed an HR of 0.79 for the NAC-CCRT group (95% CI: 0.37–1.72; *p* = 0.557) (Table 3 and Fig. 1c). In subgroup analysis by nodal staging, NPC patients with N0 disease (no cervical lymph node metastasis) and N1 disease (ipsilateral cervical lymph node involvement), NAC-CCRT and CCRT-AC had similar rates of distant metastasis (15% vs. 13% and 17% vs. 16%, respectively). In contrast, patients with N3 disease (bulky node more than 6 cm and/or positive supraclavicular node) that received NAC-CCRT had a clearly lower rate of distant metastasis than patients that received CCRT-AC [17% (5 of 29 patients) vs. 31% (5 of 16 patients), respectively; *p* = 0.455]. Moreover, the HR for NAC-CCRT in patients with N3 disease for DMFS was 0.48 (95% CI: 0.14–1.67; *p* = 0.251), when compared with CCRT-AC. However, the interaction term between nodal staging and timing of chemotherapy was not statistically significant (*p* = 0.173). For very advanced T staging, the DM rates in T4 disease were 14% in NAC-CCRT and 19% in CCRT-AC.

For locoregional recurrence-free survival (LRRFS), thirty-seven patients (14%) developed locoregional recurrence (LRR) at a median time of 34 months. Nineteen patients (24%) in the NAC-CCRT group developed more LRR, while 18 patients (10%) in CCRT-AC developed less LRR. Subgroup analysis by advanced T and N staging, the LRR rates in T4 disease were 21% in NAC-CCRT and 17% in CCRT-AC. In both N3a and N3b disease, the LRR rates were 31% in NAC-CCRT and 6% in CCRT-AC. Most of the patients who developed locoregional recurrence (65%) had stage IVA-IVB disease. The NAC-CCRT group had a significantly lower 3-year LRRFS than the CCRT-AC group (70% vs. 91%; *p* < 0.001). Even after adjustment, NAC-CCRT still showed a higher risk of LRR, with an HR of 2.18 (95% CI: 0.98–4.87; *p* = 0.057) (Table 3 and Fig. 1d). In competing-risk analysis of LRR and death, the subdistribution hazard ratio (SHR) was similar to the HR from traditional analysis by Cox proportional hazards regression.

## Discussion

In this retrospective cohort study, we set forth to investigate and compare survival outcomes between LA-NPC patients treated with CCRT-AC and those treated with NAC-CCRT. This study found that LA-NPC patients treated with NAC-CCRT had a lower 3-year and 5-year overall survival than patients treated with CCRT-AC (72% vs. 86% and 62% vs. 75%, respectively; HR: 1.68, 95% CI: 0.98–2.89; *p* = 0.061). However and importantly, after statistical adjustment by tumor stage, NAC-CCRT showed higher benefit than CCRT-AC for 3- and 5-year OS, although the difference between groups failed to achieve statistical significance (84% vs. 81% and 74% vs. 70%, respectively; HR: 0.83, 95% CI: 0.45–1.56; *p* = 0.571). These result may be explained by selection bias due to a high proportion of stage IV disease in the NAC-CCRT group. The treatment policy at our center dictates that LA-NPC patients with bulky T4 lesion and N3 disease be offered NAC-CCRT, while patients with stage II-III patients are to be offered CCRT-AC. After adjusted analysis, the 3-year estimated OS in our study (84% for NAC-CCRT and 81% for CCRT-AC) was similar to the 3-year OS (85% for NAC-CCRT and 83% for CCRT-AC) reported from a randomized controlled trial by Lee AW, et al. [14], which had the same chemoradiotherapy regimen. A network meta-analysis reported NAC-CCRT to be marginally significantly superior to CCRT-AC for overall survival, with an HR: 0.81 (95% CI: 0.61–1.07) [16]. Although the results from the present study and previous study are insufficiently robust to conclusively confirm that NAC-CCRT delivers better overall survival than CCRT-AC, the results of this study support NAC-CCRT as a viable and effective treatment option for LA-NPC, particular in patients with bulky T4 and N3 disease.

Regarding distant metastasis, distant disease control between induction chemotherapy (IC) followed by concomitant chemoradiotherapy (CRT) and CRT followed by adjuvant chemotherapy was not found to be significantly different (HR: 0.74, 95% CI: 0.43–1.25), based on the best available evidence from network meta-analysis [16]. However, a Bayesian network meta-analysis by Yu H, et al. showed that IC-CRT was marginally significant increase distant metastasis control compared with CRT (Risk ratios 1.79, 95% credible interval 0.24–1.04) [17]. To date, no large randomized control trial (RCT) has compared induction chemotherapy to adjuvant chemotherapy in a CCRT setting relative to distant metastasis outcome. Lee AW, et al. [14] reported only progression-free survival and overall survival. In our study and given the higher proportion of N3 stage disease in the NAC-CCRT group, the distant metastasis rate between NAC-AC and CCRT-AC was similar (16% in both groups). However, in subgroup analysis we found NAC-CCRT to have a lower rate of distant metastasis than CCRT-AC in N3 stage disease (HR: 0.48, 95% CI: 0.14–1.67; *p* = 0.251). Given the relatively small size of our study population, our study lacked the power to identify statistically significant differences between groups. Nevertheless, our result supports the hypothesis that patients with advanced nodal stage NPC have micrometastatic disease, and induction chemotherapy may be an indicated initial treatment in this subgroup of patients. Chua DT, et al. reported distant metastasis to be the most common pattern of failure in N3 stage NPC [13]. Xu, et al. showed that adjuvant chemotherapy significantly reduced the distant metastasis rate in N3 stage NPC patients (HR: 0.41, 95% CI: 0.19–0.88; *p* = 0.022), and also improved overall survival (HR: 0.40, 95% CI: 0.19–0.85; *p* = 0.017) [18].

Locoregional recurrence is a challenging development in nasopharyngeal carcinoma. The LA-NPC locoregional recurrence rate was 14% in this study, which was similar to an IMRT series that reported a rate of 10–15% [19]. In our study, locoregional relapse-free survival (LRRFS) was lower in the NAC-CCRT group than in the CCRT-AC group. Moreover, the LRRFS remained lower in NAC-CCRT than CCRT-AC in multivariate adjusted analysis (HR: 2.18, 95% CI: 0.98–4.87; *p* = 0.057). This our result represented the LRR problem from delay of standard local therapy due to upfront systemic therapy in the real world. A retrospective study by Wu SY, et al. reported that neoadjuvant chemotherapy followed by radiotherapy alone (NACT) delivered poorer locoregional control than concurrent chemoradiotherapy (HR: 1.75, 95% CI: 0.74–4.16; *p* = 0.2 for whole group; and, HR: 6.31, 95% CI: 1.22–32.59; *p* = 0.03 for subjects without recurrence or death in the first two years) [20]. A multi-center retrospective study using propensity score matching analysis by Song JH, et al. showed that 5-year locoregional failure-free survival (LRFFS) was statistically significant inferior in NAC-CCRT to CCRT (72% vs. 85%) [21]. These might be explained by the fact that half of the patients in those studies received two-dimensional radiotherapy. Although all patients in our study underwent IMRT and CCRT after NAC, LRR was still shown to be the main pattern of failure in neoadjuvant chemotherapy. Here are some reasons that may explain the high rate of LRR in NAC-CCRT. Firstly, the response rate to neoadjuvant TPF regimen (docetaxel, cisplatin, and 5-fluorouracil) in NPC is 90%, but the response rate to PF regimen (cisplatin and 5-fluorouracil) is lower [22]. Therefore, patients that fail to respond to chemotherapy have delayed main treatment with added toxicity. Secondly, even though most patients had response to neoadjuvant chemotherapy, delayed local treatment could become a concern. Third and last, target delineation is the most important issue, because nasopharyngeal carcinoma is a chemo-sensitive tumor with marked tumor shrinkage expected after NAC. As such, a failure to properly and completely target the tumor would increase the likelihood of LRR. Moreover, the timing of previous CT/MRI scan before starting NAC is critical. If either scan is performed too long before the start of NAC, the image may no longer accurately represent the location and scale of the tumor.

There are some plausible reasons that may explain why our study did not show adjustment survival benefit of NAC-CCRT. First, the main hypothesis of our study was reduction in distant metastasis failure in high-risk group of NPC patients by induction chemotherapy based on micrometastasis theory. Unfortunately, our induction chemotherapy patients did not show benefit for DMFS in the whole group, but we observed a lower distant metastasis rate for N3 stage NPC in NAC-CCRT with non-statistical significance. Secondly, the LRR rate in the NAC-CCRT group was significantly higher than the rate in the CCRT-RT group. Although patients with LRR were less likely to die from localized disease, LRR enhanced distant metastasis dissemination, which resultingly caused death from nasopharyngeal carcinoma [23]. A study by Lee AW, et al. [9] showed a positive correlation between locoregional control and improved long-term overall survival. To sum up, the benefit of reduction in distant metastasis rate in only high-risk subgroups by induction chemotherapy was not superior to the higher LRR rate in the whole group in our study, so no significant survival advantage was demonstrated.

This study has some mentionable limitations. Firstly, we had some inherent selection bias given that patients with T4 and/or N3 stage disease tended to receive induction chemotherapy; however, some of the bias effect was eliminated or reduced by multivariate regression analysis. Secondly, although we were able to determine vital status in all included patients, we were uncertain about the pattern of failure beyond our median follow-up of 37 months. Thirdly, chemotherapy in our practice was heterogeneous given that decision regarding cisplatin vs. carboplatin was based on kidney function and performance status. Fourthly, we don’t have any data regarding toxicity and cost which are also important for decision making between NAC-CCRT and CCRT-AC. Importantly, the strength of this study is that this data is representative of treatment outcomes in a real-world endemic setting.

## Conclusions

The results suggested that prognosis in the NAC-CCRT treated patients was not superior to that of the CCRT-AC treated individuals. In patients that receive neoadjuvant chemotherapy, locoregional relapse should be of concern. High-risk distant metastasis patients (N3 stage) that will achieve survival advantage from NAC-CCRT is an interesting and important topic for further study.

## Additional file

Additional file 1: Per-protocol analysis for overall survival, distant metastasis free survival, loco-regional relapse free survival of the study (*N* = 230 patients). (DOCX 14 kb)

## Abbreviations

CCRT: Concurrent chemoradiotherapy
CCRT-AC: Concurrent chemoradiotherapy followed by adjuvant chemotherapy
DMFS: Distant metastasis-free survival
LA-NPC: Locally advanced nasopharyngeal carcinoma
LRRFS: Locoregional relapse-free survival
NAC-CCRT: Neoadjuvant chemotherapy followed by concurrent chemoradiotherapy
OS: Overall survival

## References

[1] Pathmanathan R, Prasad U, Chandrika G, Sadler R, Flynn K, Raab-Traub N (1995). Undifferentiated, nonkeratinizing, and squamous cell carcinoma of the nasopharynx. Variants of Epstein-Barr virus-infected neoplasia. Am J Pathol.

[2] Thompson LD (2007). Update on nasopharyngeal carcinoma. Head Neck Pathol.

[3] Young LS, Dawson CW (2014). Epstein-Barr virus and nasopharyngeal carcinoma. Chin J Cancer.

[4] Shanmugaratnam K, Chan SH, de-The G, Goh JE, Khor TH, Simons MJ, Tye CY (1979). Histopathology of nasopharyngeal carcinoma: correlations with epidemiology, survival rates and other biological characteristics. Cancer.

[5] Peng H, Chen L, Zhang Y, Guo R, Li WF, Mao YP, Tan LL, Sun Y, Zhang F, Liu LZ (2016). Survival analysis of patients with advanced-stage nasopharyngeal carcinoma according to the Epstein-Barr virus status. Oncotarget.

[6] Blanchard P, Lee A, Marguet S, Leclercq J, Ng WT, Ma J, Chan AT, Huang PY, Benhamou E, Zhu G (2015). Chemotherapy and radiotherapy in nasopharyngeal carcinoma: an update of the MAC-NPC meta-analysis. Lancet Oncol.

[7] Al-Sarraf M, LeBlanc M, Giri PG, Fu KK, Cooper J, Vuong T, Forastiere AA, Adams G, Sakr WA, Schuller DE (1998). Chemoradiotherapy versus radiotherapy in patients with advanced nasopharyngeal cancer: phase III randomized intergroup study 0099. J Clin Oncol.

[8] Lee AW, Lau WH, Tung SY, Chua DT, Chappell R, Xu L, Siu L, Sze WM, Leung TW, Sham JS (2005). Preliminary results of a randomized study on therapeutic gain by concurrent chemotherapy for regionally-advanced nasopharyngeal carcinoma: NPC-9901 trial by the Hong Kong nasopharyngeal Cancer study group. J Clin Oncol.

[9] Lee AWM, Tung SY, Ng WT, Lee V, Ngan RKC, Choi HCW, Chan LLK, Siu LL, Ng AWY, Leung TW, et al. A multicenter, phase 3, randomized trial of concurrent chemoradiotherapy plus adjuvant chemotherapy versus radiotherapy alone in patients with regionally advanced nasopharyngeal carcinoma: 10-year outcomes for efficacy and toxicity. Cancer. 2017; 10.1002/cncr.30850 28662313

[10] Wee J, Tan EH, Tai BC, Wong HB, Leong SS, Tan T, Chua ET, Yang E, Lee KM, Fong KW (2005). Randomized trial of radiotherapy versus concurrent chemoradiotherapy followed by adjuvant chemotherapy in patients with American joint committee on Cancer/international union against cancer stage III and IV nasopharyngeal cancer of the endemic variety. J Clin Oncol.

[11] Lee V, Kwong D, Leung TW, Lam KO, Tong CC, Lee A (2017). Palliative systemic therapy for recurrent or metastatic nasopharyngeal carcinoma - how far have we achieved?. Crit Rev Oncol Hematol.

[12] Liao JF, Ma L, Du XJ, Lan M, Guo Y, Zheng L, Xia YF, Luo W (2016). Prognostic value of cavernous sinus invasion in patients with nasopharyngeal carcinoma treated with intensity-modulated radiotherapy. PLoS One.

[13] Chua DT, Sham JS, Wei WI, Ho WK, Au GK (2001). The predictive value of the 1997 American joint committee on Cancer stage classification in determining failure patterns in nasopharyngeal carcinoma. Cancer.

[14] Lee AW, Ngan RK, Tung SY, Cheng A, Kwong DL, Lu TX, Chan AT, Chan LL, Yiu H, Ng WT (2015). Preliminary results of trial NPC-0501 evaluating the therapeutic gain by changing from concurrent-adjuvant to induction-concurrent chemoradiotherapy, changing from fluorouracil to capecitabine, and changing from conventional to accelerated radiotherapy fractionation in patients with locoregionally advanced nasopharyngeal carcinoma. Cancer.

[15] Lee NY, Zhang Q, Pfister DG, Kim J, Garden AS, Mechalakos J, Hu K, Le QT, Colevas AD, Glisson BS (2012). Addition of bevacizumab to standard chemoradiation for locoregionally advanced nasopharyngeal carcinoma (RTOG 0615): a phase 2 multi-institutional trial. Lancet Oncol.

[16] Ribassin-Majed L, Marguet S, Lee AWM, Ng WT, Ma J, Chan ATC, Huang PY, Zhu G, Chua DTT, Chen Y (2017). What is the best treatment of locally advanced nasopharyngeal carcinoma? An individual patient data network meta-analysis. J Clin Oncol.

[17] Yu H, Gu D, He X, Gao X, Bian X (2016). The role of induction and adjuvant chemotherapy in combination with concurrent chemoradiotherapy for nasopharyngeal cancer: a Bayesian network meta-analysis of published randomized controlled trials. Onco Targets Ther.

[18] Xu T, Shen C, Ou X, He X, Ying H, Hu C (2016). The role of adjuvant chemotherapy in nasopharyngeal carcinoma with bulky neck lymph nodes in the era of IMRT. Oncotarget.

[19] Zhang B, Mo Z, Du W, Wang Y, Liu L, Wei Y (2015). Intensity-modulated radiation therapy versus 2D-RT or 3D-CRT for the treatment of nasopharyngeal carcinoma: a systematic review and meta-analysis. Oral Oncol.

[20] Wu SY, Wu YH, Yang MW, Hsueh WT, Hsiao JR, Tsai ST, Chang KY, Chang JS, Yen CJ (2014). Comparison of concurrent chemoradiotherapy versus neoadjuvant chemotherapy followed by radiation in patients with advanced nasopharyngeal carcinoma in endemic area: experience of 128 consecutive cases with 5 year follow-up. BMC Cancer.

[21] Song JH, Wu HG, Keam BS, Hah JH, Ahn YC, Oh D, Noh JM, Park HJ, Lee CG, Keum KC (2016). The role of neoadjuvant chemotherapy in the treatment of nasopharyngeal carcinoma: a multi-institutional retrospective study (KROG 11-06) using propensity score matching analysis. Cancer Res Treat.

[22] Peng H, Chen L, Li WF, Guo R, Mao YP, Zhang Y, Guo Y, Sun Y, Ma J (2017). Tumor response to neoadjuvant chemotherapy predicts long-term survival outcomes in patients with locoregionally advanced nasopharyngeal carcinoma: a secondary analysis of a randomized phase 3 clinical trial. Cancer.

[23] Kwong D, Sham J, Choy D (1994). The effect of loco-regional control on distant metastatic dissemination in carcinoma of the nasopharynx: an analysis of 1301 patients. Int J Radiat Oncol Biol Phys.

