# Supplementary material for: Neoadjuvant chemotherapy followed by concurrent chemoradiotherapy versus concurrent chemoradiotherapy followed by adjuvant chemotherapy in locally advanced nasopharyngeal carcinoma
Source: BMC Cancer. 2018 Mar 27;18:329. doi: 10.1186/s12885-018-4210-3 (PMC5870479; doi:10.1186/s12885-018-4210-3)
Supplement: Supplementary file 1 — Per-protocol analysis for overall survival, distant metastasis free survival, loco-regional relapse free survival of the study (N = 230 patients). (DOCX 14 kb) [file 12885_2018_4210_MOESM1_ESM.docx]

Additional file 1: Per-protocol analysis for overall survival, distant metastasis free survival, loco-regional relapse free survival of the study (N=230 patients)

| Event | NAC-CCRT*,  survival rate (95% CI)  (N=76 patients) | CCRT-AC**,  survival rate (95% CI)  (N=154 patients) | p-value | |
| --- | --- | --- | --- | --- |
|  | Hazard Ratio (95% CI) | | |  |
| Overall survival (OS) |  |  | |  |
| Dead, n(%) | 20 (26%) | 26 (17%) | |  |
| 3-year OS | 73% (59%-82%) | 89% (82%-93%) | |  |
| 5-year OS | 62% (45%-75%) | 78% (68%-85%) | |  |
| Univariable analysis | 2.04 (1.14-3.66) | | | 0.017 |
| Multivariable analysis† | 1.22 (0.61-2.44) | | | 0.567 |
|  |  |  | |  |
| Distant metastasis free survival (DMFS) |  |  | |  |
| Distant metastasis rate, n(%) | 13 (17%) | 24 (16%) | |  |
| 3-year DMFS | 78% (65%-87%) | 85% (78%-90%) | |  |
| 5-year DMFS | 78% (65%-87%) | 79% (69%-86%) | |  |
| Univariable analysis | 1.46 (0.74-2.87) | | | 0.276 |
| Multivariable analysis† | 1.21 (0.53-2.77) | | | 0.656 |
|  |  |  | |  |
| Loco-regional relapse free survival (LRRFS) |  |  | |  |
| Loco-regional relapse rate, n(%) | 19 (25%) | 11 (7%) | |  |
| 3-year LRRFS | 69% (56%-80%) | 94% (89%-97%) | |  |
| 5-year DMFS | 69% (56%-80%) | 88% (78%-94%) | |  |
| Univariable analysis | 4.33 (2.05-9.12) | | | <0.001 |
| Multivariable analysis† | 2.93 (1.21-7.13) | | | 0.018 |

*NAC-CCRT: neoadjuvant chemotherapy followed by concurrent chemo-radiotherapy excluded a patient who did not receive concurrent chemotherapy (3 patients as in Table 2)

**CCRT-AC: concurrent chemo-radiotherapy followed by adjuvant chemotherapy excluded a patient who did not receive adjuvant chemotherapy (33 patients as in Table 2)

† Adjusted factors: gender, age, histology type, AJCC staging, complete of radiation treatment
